# UnPAXing the Divergent Roles of PAX2 and PAX8 in High-Grade Serous Ovarian Cancer

**DOI:** 10.3390/cancers10080262

**Published:** 2018-08-08

**Authors:** Laura R. Hardy, Amrita Salvi, Joanna E. Burdette

**Affiliations:** Department of Medicinal Chemistry and Pharmacognosy, College of Pharmacy, University of Illinois at Chicago, Chicago, IL 60607, USA; lrodge4@uic.edu (L.R.H.); amritas@uic.edu (A.S.)

**Keywords:** high-grade serous ovarian carcinoma (HGSC), PAX2, PAX8, cell of origin, ovary, fallopian tube

## Abstract

High-grade serous ovarian cancer is a deadly disease that can originate from the fallopian tube or the ovarian surface epithelium. The PAX (paired box) genes PAX2 and PAX8 are lineage-specific transcription factors required during development of the fallopian tube but not in the development of the ovary. PAX2 expression is lost early in serous cancer progression, while PAX8 is expressed ubiquitously. These proteins are implicated in migration, invasion, proliferation, cell survival, stem cell maintenance, and tumor growth. Hence, targeting PAX2 and PAX8 represents a promising drug strategy that could inhibit these pro-tumorigenic effects. In this review, we examine the implications of PAX2 and PAX8 expression in the cell of origin of serous cancer and their potential efficacy as drug targets by summarizing their role in the molecular pathogenesis of ovarian cancer.

## 1. Introduction

In 2017, there were 22,440 new cases of ovarian cancer and 14,080 deaths [1]. Ovarian cancer is the fifth leading cause of cancer related death in women and the most lethal gynecological malignancy. High-grade serous carcinoma (HGSC) accounts for 80% of ovarian cancer cases and it is the deadliest histological subtype of epithelial ovarian cancer (EOC). This high mortality rate is due in part to the insidious nature of the disease, as the majority of cases are detected at an advanced stage with distant metastases. Symptoms of HGSC, such as abdominal pressure, bloating, and urinary frequency, are non-specific and do not present until after the tumor cells have metastasized and obstructed organs in the peritoneum. Current treatment strategies at this late stage include surgical debulking followed by chemotherapy with platinum and taxane drugs. While tumors are initially responsive to chemotherapy, the 5-year survival rate remains poor because of drug resistance and subsequent patient relapse. Patients with chemoresistant disease may receive chemotherapy in combination with targeted therapy against PARP (Olaparib) or VEGF-A (Bevacizumab) [2,3].

While it was originally believed that the ovary was the primary site of HGSC development, accumulating histologic, molecular, and animal model evidence suggests that the majority of cases originate from the fallopian tube epithelium [4,5,6,7]. The PAX (paired box) genes PAX2 and PAX8 are lineage-specific transcription factors that are involved in epithelial development of the fallopian tube but not the ovary [8,9]. PAX8 is expressed in HGSC tumors derived from both the fallopian tube and ovarian surface epithelium (OSE), at least in murine models where the source of the tumor is experimentally derived. In regard to the other histotypes of EOC, PAX8 shows high expression in clear cell and endometrioid tumors and reduced expression in mucinous tumors [10,11,12]. PAX2 is lost early in the molecular progression of fallopian tube derived cancer and is absent in ~85% of HGSC. PAX2 is detected in clear cell and mucinous tumors and absent in most endometrioid tumors [13,14,15,16]. Studying PAX2 and PAX8 in this context provides valuable insight into the site of origin of ovarian cancer and the tumorigenic properties that make the PAX proteins promising drug targets for treatment of HGSC.

## 2. Site of Origin of HGSC

The origin of HGSC has perplexed researchers for decades and it is now known that HGSC can originate from the fallopian tube epithelium as well as the OSE. Since PAX2 and PAX8 are expressed in the fallopian tube, and PAX8 expression is maintained in HGSC, the expression and regulation of PAX proteins may help to explain the source of ovarian cancer. The OSE was historically believed to be the site of origin of serous carcinoma based on the incessant ovulation hypothesis. This hypothesis suggests that during ovulation, fragments of the OSE get trapped within the wound created by follicle rupture, forming an ovarian cyst [17]. The epithelium trapped within the cyst has direct contact with the stroma and therefore has increased exposure to the stromal microenvironment, including growth factors and cytokines [18]. As a result, cells within an ovarian cyst have a higher likelihood of transforming into tubal-like cells that express markers of ovarian cancer, including PAX8, CA-125 and E-cadherin [18,19]. This hypothesis is supported by epidemiological data showing pregnancy and oral contraceptive use, both of which decrease the number of ovulatory cycles, are correlated with a decrease in ovarian cancer risk [20,21].

The OSE is unique to the female reproductive tract in that it is formed embryologically from the mesodermally derived colemic epithelium. In contrast, other components of the female reproductive tract, including the fallopian tube, cervix, and uterus, are Müllerian-derived structures. These Müllerian-derived structures express PAX8, while the OSE does not. This difference in embryonic origin has implications for adult cells. The adult OSE contains a mix of epithelial and mesenchymal-like cells that appear to be less differentiated than the rest of the female reproductive tract. These cells do not express molecular markers characteristic of epithelial cells, including CA-125 and E-Cadherin, but rather express mesenchymal markers, including keratin and vimentin [18]. Serous tumors that are derived from the OSE, however, obtain expression of these epithelial markers as well as phenotypic characteristics of the epithelium, including papillary serous structures [22]. Thus, in mouse models, HGSC can experimentally originate from the OSE.

The differentiated serous histology of HGSC is an interesting paradox since most cancers are less differentiated than the tissue of origin. Cheng et al. hypothesized that the OSE is an incompletely differentiated tissue type that can differentiate during oncogenic transformation through expression of *HOX* genes [23]. *HOX* genes are tightly controlled genes involved in developmental programming of the Müllerian duct, but they are not expressed in development of the OSE. This is similar to the *PAX8* gene, which is expressed in the fallopian tube and in serous tumors, but not in the OSE. By experimentally expressing *Hoxa9*, researchers observed the OSE formed serous papillary tumors. The OSE may also harbor a stem cell niche within the transitional zone of the ovarian hilum that has increased tumorigenic properties. Researchers experimentally demonstrated that cells within the ovarian hilum express stem cell markers that contribute to regeneration of the OSE [24]. Importantly, these stem cells had greater transformative ability after conditional inactivation of *p53* and *RB1*. It would be interesting to examine whether these stem cells also gained developmental markers, such as HOXA9 or PAX8 that would induce differentiation to a serous histotype.

Increasing evidence indicates that the fallopian tube epithelium serves as the main site of origin of HGSC. Under this scenario, serous tumors found on the ovarian surface are secondary metastasis from the fallopian tube, and thus resemble this lineage history. Piek et al. presented the first clinical evidence supporting this hypothesis by identifying pre-neoplastic lesions with increased staining for p53 and Ki67 in the fallopian tubes of BRCA-positive women who are predisposed to developing ovarian carcinoma [5]. Identical p53 mutations were identified in the precursor lesions of the fallopian tube and in concurrent ovarian carcinomas [6,25]. Molecular profiling of serous tumors identified a gene signature in HGSC tumors that more closely correlated with the normal fallopian tube epithelium than the normal OSE [26,27]. Clinically, bilateral salpingectomy reduced the risk of serous carcinoma by 61% and prophylactic salpingo-oophorectomy in BRCA-positive women reduced the risk of serous carcinoma by 80% [28,29]. Therefore, the current recommendation states that BRCA-positive women after child bearing age should undergo prophylactic salpingo-oophorectomy [30].

The fallopian tube origin for ovarian cancer is further supported by multiple animal models. Immortalized fallopian tube secretory epithelial cells are transformed into HGSC through *H-Ras^V12^* mutation or *c-Myc* expression [31]. *Dicer-Pten* deletion from the reproductive tract resulted in HGSC formation, even after bilateral removal of the ovaries, demonstrating that these tumors originated in the fallopian tube [7]. *Pax8* promoter-driven deletion of *Brca*, *Tp53*, and *Pten* in the fallopian tube also led to HGSC development [32]. Since a common molecular alteration in these models is loss of *Pten*, Russo and colleagues examined the effects of *Pten* loss alone from the fallopian tube epithelium. Homozygous loss of *Pten* was sufficient to drive the development of borderline serous and endometrioid carcinoma that could metastasize to the ovary [33]. Interestingly, in a cell-based model, *Pten* loss in combination with *Kras* mutation formed highly aggressive tumors, while addition of constitutively active *Akt* attenuated this phenotype [34]. Research from the Cho laboratory demonstrated how serous carcinoma progresses from serous tubal intraepithelial carcinoma (STIC) to HGSC using various combinatorial deletions in *Rb1*, *Brca1*, *p53*, *Nf1* [35]. These tumor models derived from the fallopian tube epithelium provide researchers with the tools to study the molecular progression from pre-neoplastic lesion to aggressive serous carcinoma.

Careful examination and sequencing of patients with HGSC paints a more nuanced picture of the cell of origin debate. Laser-capture tumor microdissection of multiple anatomic sites in patients with HGSC showed an identical *p53* mutation at all sites [36]. The metastatic trajectory of HGSC was elucidated using phylogenetic clustering that compared tumor mutations to a patient’s germline DNA. While the majority of patient tumors clustered in the “basal STIC” category, with the STIC showing the highest similarity to germline DNA, some tumors showed “STIC metastases”. These findings call into question the assumption that the presence of STICs is always evidence for a fallopian tube origin for HGSC. A separate evolutionary analysis study that sequenced STICs, ovarian cancer, and metastases in nine patients found tumor-specific alterations in p53, BRCA1, BRCA2, or PTEN to be present in STICs [37]. This finding implies that in the majority of cases, mutations that drive HGSC occur early, before metastasis to the ovary. In a proteomic study of HGSC cell lines and patient tumor samples, 26 ovarian cancer cell lines and five HGSC tumors were grouped into three distinct categories: epithelial, clear cell, and mesenchymal [38]. While most cell lines and tumors in this study clustered in the epithelial group, suggesting a fallopian tube cell of origin, the authors identified a subset of cell lines and one HGSC tumor that grouped in the mesenchymal category, suggesting an ovarian cell of origin [38]. This demonstrates HGSC may arise from both the fallopian tube and OSE or that cells may acquire markers during tumorigenesis that resemble different tissues.

PAX2 and PAX8 are expressed in the fallopian tube epithelium, however, PAX2 is lost in ~85% HGSC and it has been shown that mutant p53 and loss of PTEN represses PAX2 expression in a fallopian tube-derived mode of ovarian cancer [39]. On the contrary, PAX8 is expressed in 85–90% of HGSC and is a widely used biomarker for HGSC [4,16,40]. PAX2 and PAX8 are differentially regulated in HGSC and it will be interesting to know whether loss of PAX2 during HGSC progression leads to dependence of HGSC on PAX8. Thus, studying the shared regulatory mechanisms of PAX2 and PAX8 expression between the fallopian tube and ovary will be essential to developing effective treatment therapies until the site of origin of a patient’s tumor can be definitively identified.

## 3. Role of PAX2 and PAX8 in Development and Adult Tissues

The *PAX* genes are a set of developmental transcription factors that are key regulators for proper tissue formation and cellular differentiation [41]. This is convincingly supported by mouse models with *Pax* gene deletions. PAX2 is required for mesenchymal-to-epithelial transition of the intermediate mesoderm into the epithelial structures of the inner ear, kidneys, ureters, Wolffian and Müllerian ducts, including the oviducts, uterus, and vagina [42]. Mice with *Pax2* homozygous mutation do not develop these structures. Research has shown that *Pax2* is a tissue-specific epigenetic regulatory gene that ensures proper temporal and spatial development of these epithelial structures. The Hashino laboratory demonstrated that in progenitor cells of the inner ear, the histone demethylase LSD1 recruits the NuRD co-repressor complex to bind and repress PAX2 target genes. This inhibition ensures tight temporal control of PAX2-regulated genes. Once cells enter the differentiated state to become epithelial cells, LSD1 and the NuRD complex are released from the PAX2 binding site, and transcription can occur. This switch from progenitor intermediate mesoderm to differentiated epithelium is irreversible and is maintained over rapidly dividing cell populations through PAX2-regulated epigenetic modifications [43]. Research from the Dressler laboratory demonstrated that PAX2 promotes assembly of the histone H3K4 methylation complex by recruiting PTIP (PAX transcription activation domain interacting protein) at PAX2 binding elements [44]. This histone modification is associated with active promoters and increased transcription. PTIP deletion inhibits histone H3K4 methylation, even though PAX2 still binds to the chromosome. These data suggest that PTIP regulates epigenetic modifications required for activation of PAX2 targets that are essential for development and maintenance of epithelial structures.

PAX2 expression persists in adult reproductive tissues (epididymis, vas deferens, oviduct), ureters, bladder, kidneys, and mammary glands [45]. Cai et al. demonstrated that PAX2 levels are osmotically regulated [46]. Exposing medullary epithelial cells in vitro to high levels of NaCl increased PAX2 levels, while reducing in vivo renal inner-medullary interstitial NaCl levels decreased PAX2 levels. This increase in PAX2 appears to protect against cell death induced by osmotic stress. The stem cells of the mammary duct also express PAX2 where it may protect against apoptosis [47]. This is supported by research in *C. elegans* which demonstrates PAX2/5/8 can upregulate transcription of the anti-apoptotic *Bcl2* [48].

PAX8 is a closely related paralog to PAX2 that is expressed during embryogenesis in the thyroid, metanephros, central nervous system, and Müllerian duct. Inactivation of the *Pax8* gene in mice leads to complete loss of thyroid follicular cells, severe growth retardation, and death in the perinatal period [49]. Providing exogenous thyroid hormone to *Pax8*^−/−^ mice rescued the hypothyroid phenotype, but these mice remained infertile due to nonfunctional uteri and closed vaginal openings [50].

PAX8 continues to be expressed in the adult kidneys, cervix, endometrium, fallopian tube, seminal vesicle, epididymis, thyroid, pancreas, and lymphoid cells [10,51]. There is also evidence that a subset of cells in the OSE express PAX8, but further research will need to examine the mechanism for this acquired expression [51,52]. The majority of our understanding of PAX8 function in adults is based on studies in the thyroid. Zannini and colleagues demonstrated that PAX8 is required for expression of the thyroid-specific genes: thyroglobulin, thyroperoxidase, and sodium/iodide symporter [53,54]. Interestingly, ChIP-Seq demonstrated PAX8 tends to bind in intronic regions (82%) over 5’-UTR regulatory regions (2%) [55]. This suggests PAX8 may bind alternative promoters or ncRNAs that regulate gene expression. Additionally, immunoprecipitation studies demonstrated that PAX8 binds CTCF and SP1, both of which are involved in chromatin remodeling [55]. These data suggest PAX8 functions both to directly increase transcription and to remodel the chromatin landscape.

## 4. Role of PAX2 and PAX8 in HGSC

Examining the histologic and molecular events that give rise to serous carcinoma is crucial to understanding the drivers of ovarian cancer. Secretory cell outgrowths (SCOUTs) are precursor lesions of serous carcinoma that can be found in the proximal and distal fallopian tube. Normal fallopian tube epithelial cells express high levels of PAX2 but approximately 90% of SCOUTs have lost PAX2 expression [16]. Almost all serous tumor cells also have mutation in the tumor suppressor p53, yet only 25% of SCOUTs have p53 mutation that can be detected histologically [16,56]. SCOUTs located at the fimbrial edge with p53 mutation are coined ‘p53 signatures’ [16]. Cells with the p53 signature have PAX2 loss, suggesting a step-wise progression from PAX2 loss to p53 signature to STIC to metastatic serous carcinoma. This progression has been extensively researched, and there are many excellent reviews detailing these findings [4,30,57,58].

**Figure 1:**
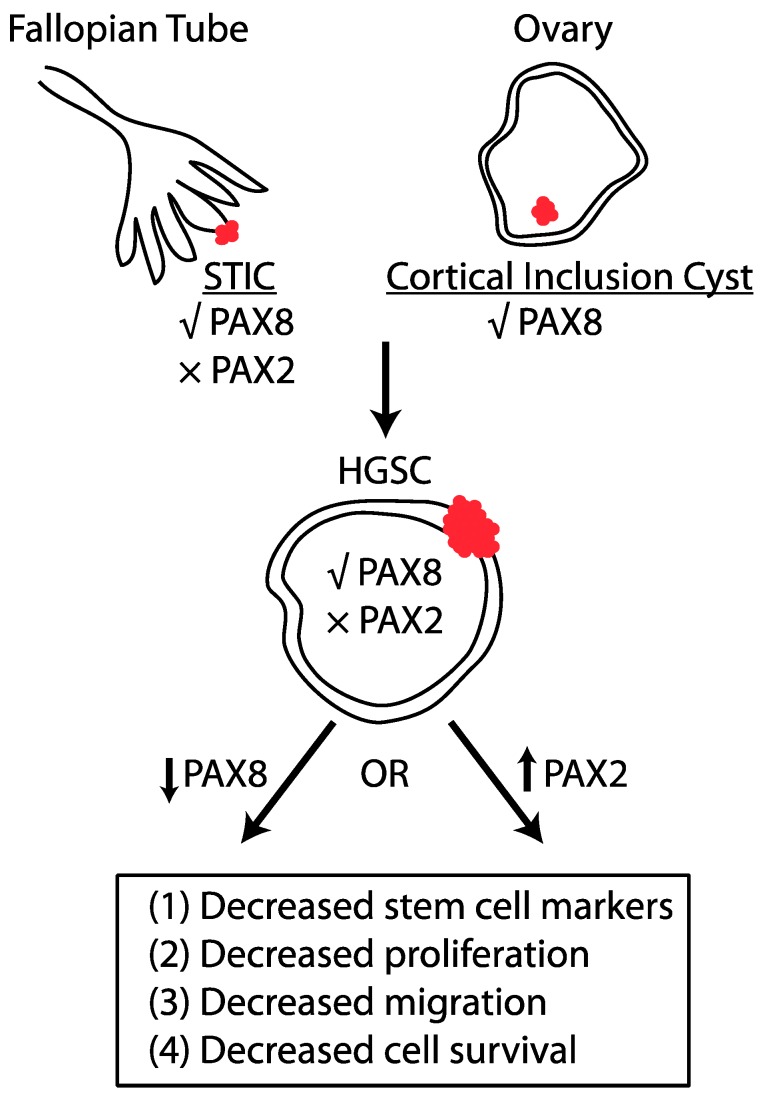
PAX2 and PAX8 regulate tumor formation in HGSC in an opposing manner. Serous tubal intraepithelial carcinomas (STICs) express PAX8, but not PAX2. Similarly, epithelial cells in cortical inclusion cysts express PAX8. HGSC tumor cells express PAX8 and it has been experimentally shown that PAX8 reduction decreases characteristics that enhance tumor formation. PAX2 is not expressed in HGSC and re-expression of PAX2 inhibits the tumorigenic properties of tumor cells.

Through molecular characterization of SCOUTs, Ning and colleagues demonstrated that PAX2 loss is associated with an increased stem cell phenotype [59]. They show through in vitro culture of SCOUTs that these cells can differentiate into both ciliated and basal cell histotypes. PAX2 knockdown in fallopian tube epithelial cell lines increased expression of the stem cell markers CD44 and SCA1 and decreased the capability of these cells to form differentiated epithelial luminal structures [60]. Modi et al. demonstrated in murine oviductal epithelial cells that *Pax2* loss and *p53* mutation increased proliferation and migration, but was insufficient to drive tumorigenesis [39]. This is consistent with human histological findings that p53 signatures are benign secretory outgrowths. ChIP analysis revealed wild type *p53* enhances *Pax2* transcription while mutant *p53* decreases *Pax2* transcription, suggesting a mechanism for sustained *Pax2* loss in neoplastic lesions [39]. Interestingly, cells lost *Pax2* expression in a fallopian tube model of ovarian cancer derived through loss of *Pten* [39]. Re-expression of *Pax2* inhibited the tumorigenic properties of these cells and prolonged survival (Figure 1). Alternatively, *Pax2* expression in a spontaneous OSE derived model of HGSC (called STOSE) reduced proliferation and metastasis by increasing COX2 and reducing HTRA1 expression [61]. Taken together, these findings suggest *Pax2* loss is an early molecular event in ovarian cancer progression that predisposes cells to further mutations that can drive tumorigenesis, regardless of cell of origin. Further research should examine the mechanistic requirement for *Pax2* loss in HGSC progression, especially considering that there is increased hypomethylation and activation of *Pax2* in endometrial and renal carcinoma, yet The Cancer Genome Atlas (TCGA) does not find increased methylation at this locus in HGSC tumor samples [47,62,63].

Pathologists have used PAX8 for decades as a histologic marker to define HGSC, but a genome-wide RNA interference screen of cancer cell lines was the first to identify the importance of PAX8 in ovarian cancer [64]. PAX8 was the top-ranked differentially expressed gene in the screen between ovarian and non-ovarian cancer cell lines. *PAX8* knockdown reduced proliferation, migration and invasion and increased apoptosis in ovarian cancer cells [65]. *Pax8* was shown to directly bind and increase the transcription of *p53*, which then increased *p21* to induce proliferation [66]. *Pax8* also promoted tumor cell growth by increasing transcription of the cell cycle regulator *E2f1* through direct binding to the *E2f1* promoter in a complex with the RB protein [67]. In thyroid follicular carcinoma, a translocation event results in *PAX8-PPARγ1* fusion [68], but this genetic event is not observed in HGSC (regulation of PAX2 and PAX8 in specific cancers is summarized in Table 1). To better understand the mechanism of PAX8 oncogenesis in HGSC despite its normal expression in the fallopian tube, several research groups have examined the role of PAX8 in the ovary and fallopian tube. Serial passaging of the normal OSE transforms cells into serous carcinoma with PAX8 expressed [22]. Loss of LKB1 and PTEN in the OSE also leads to a HGSC cell line with acquired PAX8 expression [69]. Rodgers and colleagues demonstrated that forced PAX8 expression in normal OSE increases proliferation, migration, and epithelial-mesenchymal transition through upregulation of the FOXM1 pathway [70]. Correspondingly, *PAX8* knockdown in three human HGSC cell lines decreased expression of FOXM1, decreased proliferation, and increased apoptosis [70]. Reducing PAX8 expression in the normal fallopian tube, however, did not produce noticeable phenotypic effects, suggesting that targeting PAX8 pharmacologically would not affect normal tissues. These phenotypic observations were corroborated by Elias and colleagues who performed an RNA sequencing experiment demonstrating few transcripts altered in the fallopian tube but increased transcript alterations in serous tumors after *PAX8* knockdown. The authors suggest alterations to the *PAX8* cistrome are responsible for changes in gene expression leading to HGSC derived from the fallopian tube. The *PAX8* consensus binding motif is altered between the fallopian tube and serous tumor cells that may affect downstream regulated genes. Elias et al. show differential association between PAX8 and Yes-associated protein (YAP1), a major downstream regulator of the evolutionarily conserved Hippo pathway that regulates organ size, cell proliferation, and apoptosis [71]. Interestingly, ChIP-Seq identified PAX8 mostly binds at non-promoter sites and is enriched at super-enhancers, where PAX8 can globally regulate genes involved in tumorigenesis [72]. Taken together, these findings suggest PAX8 could be targeted for drug development to reduce proliferation, migration and survival of tumor cells while leaving other organs unaffected (Figure 1).

## 5. Clinical Strategies to Target PAX2 and PAX8

Ovarian cancer is a heterogeneous disease with few common molecular alterations [56]. Developing therapeutic strategies that target common molecular alterations, such as loss of PAX2 or gain of PAX8, may produce greater therapeutic benefits. A promoter activation screen identified luteolin as a small molecule that restores PAX2 expression in cells with wild type p53 [39]. Luteolin could be taken as a preventative supplement to decrease the occurrence of SCOUTs, but it would be ineffective in treating serous tumors with p53 mutation. Further screens or combination therapy studies should be performed in HGSC cells to identify molecules that increase PAX2 in tumors. The effect of these molecules on the homologue PAX8 should also be explored. Molecules that increase expression of PAX2 may also increase expression of PAX8, which could then increase the aggressive properties of a tumor cell. Therapies that reduce transcription of these PAX proteins, however, may significantly mediate the deleterious effect of PAX8 while maintaining the already decreased PAX2 levels.

PAX8 seems to have little functional effect in the fully differentiated adult fallopian tube, but mediates several tumorigenic effects in HGSC, including proliferation, migration, angiogenesis, and apoptosis [65,70,71,72,79]. Reducing PAX8 levels or disrupting the transcriptional activity of PAX8 may inhibit these pro-cancerous effects while leaving the normal fallopian tube epithelium unaffected. Using a virtual screen that modeled paired domain binding to DNA, Grimley and colleagues identified small molecules that disrupt binding of the paired domain of PAX2/5/8 to DNA [80]. Other potential drug targets include the adapter proteins that bind to the chromosome in a complex with PAX8. PAX8 requires interactions with YAP1, CTCF and SP1 to initiate transcription, as discussed earlier. Disrupting these interactions may mediate the deleterious effects of PAX8 in serous carcinoma.

## 6. Concluding Remarks

Proper temporal and spatial expression of the PAX protein family is essential for embryonic development. PAX2 and PAX8 are co-expressed during mesenchymal-to-epithelial transition of the Müllerian duct and they continue to be expressed in adult structures, such as the fallopian tube. These proteins maintain a regenerative stem cell population in adult tissues. In HGSC, PAX8 provides growth advantages by enhancing the proliferative, migratory, and survival capabilities of cancer cells from the fallopian tube and ovary. The OSE does not normally express PAX8, yet it acquires PAX8 expression during malignant transformation in certain mouse models. More work is required to tease apart the role of PAX8 in tumors derived from the fallopian tube or OSE. PAX2 is a homolog of PAX8 that has been shown to impart similar growth advantages, yet tumors derived from the fallopian tube epithelium lose PAX2 expression during malignant transformation. Further research is required to understand the importance and regulatory machinery that leads to PAX2 loss and PAX8 dependence in HGSC.

Identifying drug targets for novel cancer treatments in HGSC has been challenging because it is a heterogeneous disease with few shared mutations. The PAX proteins are promising because PAX8 is ubiquitously expressed in serous tumors and PAX2 loss is an early molecular event shared in the progression from benign to malignant carcinoma. Targeting these proteins may hold promise in reducing tumor growth and progression in a majority of patients and significantly improving patient survival.

## Figures and Tables

**Table 1:** Mechanism of PAX2 and PAX8 regulation in specific cancer types.

Cancer Type	PAX2 Regulation	PAX8 Regulation	References
HGSC	Transcriptional downregulation	No change	[39]
Endometrial	Promoter hypomethylation	No change	[62]
Thyroid	No change	PAX8-PPARγ1 fusion	[68]
Renal	Promoter hypomethylation	Increased protein levels	[63,73]
Wilms tumor	Transcriptional upregulation	Transcriptional upregulation	[74,75]
Breast	Transcriptional upregulation	No change	[76]
Glioma	Transcriptional upregulation	Transcriptional upregulation	[77,78]
